# Describing complex cells in primary visual cortex: a comparison of context and multifilter LN models

**DOI:** 10.1152/jn.00916.2017

**Published:** 2018-05-02

**Authors:** Johan Westö, Patrick J. C. May

**Affiliations:** ^1^Department of Neuroscience and Biomedical Engineering Aalto University, Espoo, Finland; ^2^Department of Psychology, Lancaster University, Lancaster, United Kingdom

**Keywords:** complex cell, context model, LN model, receptive field, stimulus-response mapping

## Abstract

Receptive field (RF) models are an important tool for deciphering neural responses to sensory stimuli. The two currently popular RF models are multifilter linear-nonlinear (LN) models and context models. Models are, however, never correct, and they rely on assumptions to keep them simple enough to be interpretable. As a consequence, different models describe different stimulus-response mappings, which may or may not be good approximations of real neural behavior. In the current study, we take up two tasks: *1*) we introduce new ways to estimate context models with realistic nonlinearities, that is, with logistic and exponential functions, and *2*) we evaluate context models and multifilter LN models in terms of how well they describe recorded data from complex cells in cat primary visual cortex. Our results, based on single-spike information and correlation coefficients, indicate that context models outperform corresponding multifilter LN models of equal complexity (measured in terms of number of parameters), with the best increase in performance being achieved by the novel context models. Consequently, our results suggest that the multifilter LN-model framework is suboptimal for describing the behavior of complex cells: the context-model framework is clearly superior while still providing interpretable quantizations of neural behavior.

**NEW & NOTEWORTHY** We used data from complex cells in primary visual cortex to estimate a wide variety of receptive field models from two frameworks that have previously not been compared with each other. The models included traditionally used multifilter linear-nonlinear models and novel variants of context models. Using mutual information and correlation coefficients as performance measures, we showed that context models are superior for describing complex cells and that the novel context models performed the best.

## INTRODUCTION

Receptive field (RF) models have been an important tool for discovering how the brain encodes and processes sensory information (Spillmann 2014; Spillmann et al. 2015). The goal of these models is to capture essential features of stimulation to which the cell responds and to convey this information in an interpretable manner to the scientist. However, the traditional linear-nonlinear (LN) model, defined as a single filter that is cross-correlated with the stimulus and then followed by a nonlinearity (see Ringach 2004 for a review), often achieves only the latter of these two goals. Several cells exhibit nonlinearities that cannot be described using this simple model. Examples of such cells include complex cells in visual areas (Adelson and Bergen 1985; Chen et al. 2007; Rust et al. 2005; Touryan et al. 2002), auditory cells responsive to frequency sweeps (Andoni and Pollak 2011), or specific combinations of frequencies (Brimijoin and O’Neill 2010; Brosch and Schreiner 2000; Sadagopan and Wang 2009; Schneider and Woolley 2011), and cells that exhibit effects of synaptic depression (Abbott and Regehr 2004; Abbott et al. 1997; Reyes 2011; Tsodyks and Markram 1997). Thus, there is a need for more complex models that can extend the traditional LN model to also describe these types of nonlinear behaviors. However, added complexity easily leads to models that may be difficult to interpret, and more complex models are also plagued by large numbers of parameters that need to be estimated and by subsequent requirements to obtain more data. Various extensions of the traditional LN model solve these problems by making simplifying assumptions about the cell’s nonlinearities, but at the cost of restricting which stimulus-response mappings that can be described. Consequently, one extension might be better than others if it includes unique stimulus-response mappings that better approximate the cell’s real behavior. It follows that comparing the performance of different extensions should provide more insight than using any single extension alone, because a comparison will reveal which of the underlying assumptions is the more correct one.

In this study, we compare models from two separate extension frameworks that both have proven useful for describing a range of nonlinear behaviors: The first framework is that of context models (Ahrens et al. 2008; Westö and May 2016; Williamson et al. 2016) and the second is that of multifilter LN models (de Ruyter Van Steveninck and Bialek 1988; Samengo and Gollisch 2013; Schwartz et al. 2006), with low-rank quadratic-nonlinear (QN) models included as a special case (Fitzgerald et al. 2011; Park et al. 2013; Rajan et al. 2013). Importantly, these two frameworks make different simplifying assumptions. The context model framework assumes that neural behavior is mainly influenced by an RF and a small number of stimulus contexts (not to be confused with extraclassical RF effects Spillmann et al. 2015), whereas the multifilter LN model framework assumes that only stimuli which inhabit a relevant low-dimensional stimulus subspace affect neural behavior.

The behavior of real cells is unlikely to match perfectly the stimulus-response mappings found in either framework, but one framework is likely to provide better approximations than the other. However, to our knowledge, a comparison of framework performance has not yet been carried out in the case of context models and multifilter LN models (but see Williamson et al. 2016). Using simulated data, we recently showed that the context model framework is superior when *1*) the effects of synaptic depression are present or *2*) when inputs with identical but spatially/spectrally shifted RFs drive an output cell (Westö and May 2016). These are two conditions that seem to be met by complex visual cells (Abbott et al. 1997; Hirsch and Martinez 2006; Martinez and Alonso 2003). Hence, in this study, we present a novel general method for constructing different types of context models from data. Using data from complex cells (Dan et al. 2009) and performance measures based on mutual information and correlation coefficients, we then compare the performance of these context models with that of multifilter LN models. In the future, it would be interesting to extend the analysis to include subunit models (Eickenberg et al. 2012; Vintch et al. 2012). These resemble context models and have previously been compared with multifilter LN models (Vintch et al. 2015). Nonetheless, in this first comparison, we restrict ourselves to comparing context and multifilter LN models.

## MATERIALS AND METHODS

Given a data set (*y_i_* and **x**_i_, *i* = 1,…, *N*) consisting of spike counts (*y_i_*) and input/stimuli (**x***_i_*), the aim is to extract the stimulus-response mapping (that is, the RF model) in such a way that neural responses to arbitrary stimuli can be predicted. Formally, this is achieved by finding an RF model, which is able to predict cellular responses (${\hat{y}}_{i}$) from a similarity score vector (**z***_i_*) and a nonlinearity (*f*) as

y^i=f(zi),

where **z***_i_* can be either one-dimensional or multidimensional and expresses the similarity between the stimulus **x***_i_* and the RF model. The model is derived on the basis of the stimuli **x***_i_* and spike counts *y_i_*, so that the predicted responses ${\hat{y}}_{i}$ are as close to the actual responses *y_i_* as possible. We estimate models from both the context- and the multifilter LN model frameworks and compare these to each other. An outline of the data, the models, the model estimation procedures, and the model evaluation methods are presented below, and a GitHub repository for estimating all the different models is available at receptive-field-models.

### Data

**Fig. 1.**
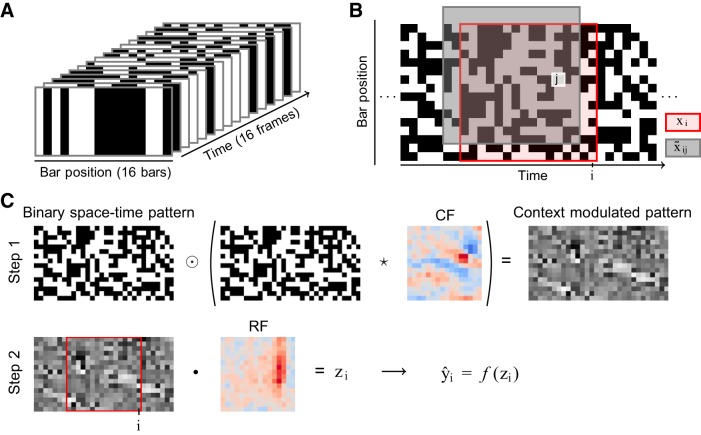
The stimulus can be viewed as a binary space-time pattern on which a context field (CF) highlights regions with the correct local context. *A*: each input vector (**x***_i_*) contains the values (dark vs. bright) of 16 bars in a time window containing 16 frames, and hence, it represents one direction in a 256-dimensional stimulus space. *B*: the whole stimulus can, thus, be visualized as a binary space-time pattern, where each column represents one frame. Input vectors are then extracted by sliding a 16-by-16 window over the stimulus, as exemplified by the highlighted region representing the space-time pattern of **x***_i_*. For each element *x_ij_*, we additionally define an input context vector ${\overset{˜}{x}}_{ij}$ corresponding to the pattern localized around that element. Each input context vector is, therefore, identified with two indices (*i* and *j*). *C*: the context model scales each input element *x_ij_* by its local context ${\overset{˜}{x}}_{ij}^{⊺}w^{\text{cf}}$ and treats the resulting context-modulated pattern as input to a traditional linear-nonlinear (LN) model. Its operation can, therefore, be visualized as occurring in two steps. In *step 1*, the original space-time pattern is element-wise (⊙) multiplied with a cross-correlation (⋆) of the pattern and the CF. The resulting context-modulated pattern is then fed as input to a traditional LN model in *step 2*, by sliding a window over it. The model similarity score *z_i_* at each time point *i* is, thus, obtained as the dot product (•) between the window and the receptive field (RF).

The data set comprised recordings from complex visual cells and was obtained from the Collaborative Research in Computational Neuroscience (CRCNS) program (Dan et al. 2009). A detailed description of how the data were collected is given by Touryan et al. (2002). In short, stimulus-response data for 61 complex cells was measured in extracellular recordings in cat striate cortex using binary pseudo-random bar stimuli aligned along the cell’s preferred orientation. Each stimulus frame consisted of 16 pseudo-random bars, and these were presented using a frame rate of 60 Hz. For each time step, we created input vectors (**x***_i_*) from the stimuli presented during the past 267 ms. As illustrated in Fig. 1, *A* and *B*, this meant that each input vector contained the pseudo-random bar patterns presented during the previous 16 frames. Consequently, each input vector can be viewed as a 16 × 16 space-time pattern or as a direction in the 256-dimensional stimulus space.

### Context Models

Context models have a single RF, but unlike traditional LN models, they also have one or more CFs. The similarity score is, hence, calculated from an RF-CF combination (Ahrens et al. 2008; Westö and May 2016; Williamson et al. 2016), where the CFs determine the conditions under which the structure in the RF influences the cell’s response. For the baseline case with one CF, the similarity score is given by

$$z_{i}=w_{0}^{\text{rf}}+\underset{j}{\sum }w_{j}^{\text{rf}}x_{ij}\left(1+\underset{k}{\sum }w_{k}^{\text{cf}}{\overset{˜}{x}}_{ijk}\right),$$

where **w** is a vectorized field indexed by *j* and *k* for the RF and CF, respectively, *x_ij_* is the element *j* in the vectorized input **x***_i_*, and ${\tilde{x}}_{ijk}$ is element *k* in the vectorized input context ${\overset{˜}{x}}_{ij}$ (see Fig. 1*B*). As illustrated in Fig. 1*C*, this means that a single-CF context model can be visualized as a traditional LN model operating on a stimulus sequence that has first been cross-correlated with the CF. In this sense, the context model is very similar to subunit models (Eickenberg et al. 2012; Vintch et al. 2012, 2015; Wu et al. 2015), which also cross-correlate (or convolve) the input with one or more filters. The cross correlation can also be interpreted, such that each RF element is scaled by the dot product between the CF and the local input around the RF element. That is, local context determines to which degree the structure in the RF influences the firing of the cell. Removing the CF, therefore, reverts the similarity score to the linear definition used in traditional LN models.

Context models can be further extended to include multiple CFs. In such cases, we need to indicate which RF elements are associated with which CF. We index contexts here by *l* = 1,…, *N*_ctx_, and we use the notation *j* ∈ ctx*_l_* to refer to all RF indices associated with context *l*. We note for later use that the similarity scores for multiple contexts (identified by the superscripts rf and cf, respectively) can be expressed in two different ways:

(1) $$z_{i}^{\text{rf}}=w_{0}^{\text{rf}}+\underset{l}{\sum }\underset{j\in {\text{ctx}}_{l}}{\sum }w_{j}^{\text{rf}}x_{ij}\left(1+\underset{k}{\sum }w_{k}^{{\text{cf}}_{l}}{\overset{˜}{x}}_{ijk}\right),$$

(2) $$z_{i}^{\text{cf}}=w_{0}^{\text{rf}}+\underset{j}{\sum }w_{j}^{\text{rf}}x_{ij}+\underset{l}{\sum }\underset{k}{\sum }w_{k}^{{\text{cf}}_{l}}\underset{j\in {\text{ctx}}_{l}}{\sum }w_{j}^{\text{rf}}{\overset{˜}{x}}_{ijk}x_{ij}.$$

### RF/CF Estimation

RF model parameters are found by minimizing a loss function that provides a measure of the deviation between predicted and actual responses. This optimization problem can often be formulated as a convex problem for traditional LN models, meaning that the loss function has one unique minimum, which then is also the global minimum. However, the inclusion of CFs makes the relevant loss functions nonconvex and more difficult to solve. To overcome this hurdle, Ahrens et al. (2008) proposed an approach in which the optimization algorithm alternates between finding the optimal RF and the optimal CFs, while keeping the CFs and the RF, respectively, temporarily fixed (see *Algorithm 1*). The resulting subproblems can then be formulated as convex linear regression problems, assuming that the nonlinearity is the identity function. We expand upon this below and show that the subproblems can be formulated as logistic or Poisson regression problems, which, while still being convex, result in more realistic nonlinearities (i.e., a logistic function or an exponential function).

**Table:** 

**Algorithm 1:** Alternating optimization algorithm for estimating context models
1: **data** **x**_i_ and *y_i_*, *i* = 1,..., *N* 2: **parameters** **w**_rf_ and $w_{{\text{cf}}_{l}},l=1,...,N_{\text{ctx}}$ 3: **while** not converged **do** 4: find the RF, **w**_rf_, while keeping CFs fixed 5: find the CFs, $w_{{\text{cf}}_{l}}$, while keeping the RF fixed 6: **return** **w**_rf_ and $w_{{\text{cf}}_{l}}$

The specific structure of the context model’s similarity score lets us simplify *Eqs. 1* and *2* by noting that the partial derivatives with respect to each field’s parameters are

$$\frac{\partial z_{i}}{\partial w_{j}^{\text{rf}}}=x_{ij}\left(1+\underset{k}{\sum }w_{k}^{{\text{cf}}_{l}}{\overset{˜}{x}}_{ijk}\right),$$

$$\frac{\partial z_{i}}{\partial w_{k}^{{\text{cf}}_{l}}}=\underset{j\in {\text{ctx}}_{l}}{\sum }w_{j}^{\text{rf}}{\overset{˜}{x}}_{ijk}x_{ij}.$$

Inserting these expressions into *Eqs. 1* and *2* gives us representations for the similarity score that are linear in the RF parameters when the CF parameters are assumed fixed, and vice versa. In vector notation, these equations become

$$z_{i}^{\text{rf}}=w_{0}^{\text{rf}}+w_{\text{rf}}^{⊺}\nabla_{w_{\text{rf}}}(z_{i}),$$

$$z_{i}^{\text{cf}}=w_{0}^{\text{rf}}+w_{\text{rf}}^{⊺}x_{i}+\overset{N_{\text{ctx}}}{\underset{l}{\sum }}w_{{\text{cf}}_{l}}^{⊺}\nabla_{w_{{\text{cf}}_{l}}}(z_{i}),$$

where $\nabla_{w}(z_{i})$ denotes the gradient of *z_i_* with respect to **w**. If we further concatenate all CFs, we arrive at a canonical representation for both equations:

(3) $$z_{i}=w_{0}+k_{i}+w^{⊺}\nabla_{w}(z_{i}),$$

where the bias term (*w*_0_) and the constant (*k_i_*) are given in Table 1 for both the RF and the CF cases. *Algorithm 1* will, therefore, alternate between finding optimal parameter values for *f*(*z*^rf^) and *f*(*z*^cf^), where *z* in both cases can be described as a linear function of its parameters. Formulating these subproblems as linear, logistic, or Poisson regression problems, therefore, corresponds to assuming that the nonlinearity is the identity function, the logistic function, or the exponential function, respectively. The loss function is then in each case given by

(4) $$L^{\text{LinReg}}=C\underset{i}{\sum }{\left(y_{i}-z_{i}\right)}^{2}+\frac{1}{2}||\Gamma w{||}^{2},$$

(5) $$L^{\text{LogReg}}=C\underset{i}{\sum }\beta_{i}\log[1+e^{-{\overset{˜}{y}}_{i}z_{i}}]+\frac{1}{2}||\Gamma w{||}^{2},$$

(6) $$L^{\text{PoiReg}}=C\underset{i}{\sum }e^{z_{i}}-y_{i}z_{i}+\frac{1}{2}||\Gamma w{||}^{2},$$

**Table 1.** Canonical bias term and constant for $z_{i}^{\text{rf}}$ and $z_{i}^{\text{cf}}$

	*w*_0_	*k_i_*
$z_{i}^{\text{rf}}$	$w_{0}^{\text{rf}}$	0
$z_{i}^{\text{cf}}$	0	$w_{0}^{\text{rf}}+w_{\text{rf}}^{⊺}x_{i}^{\text{rf}}$

where $||\Gamma w{||}^{2}$ is a Tikhonov regularization term used to avoid overfitting (to which we will return later), *C* is a regularization parameter, ${\overset{˜}{y}}_{i}$ are signed *y* values, and *β_i_* are sample-specific weights. Logistic regression is normally a classifier (spike/no spike), and we have, therefore, introduced *β_i_* to capture the effects of higher spike counts. This is done by counting samples with multiple spikes and multiple times, and, hence, we define ${\tilde{y}}_{i}$ and *β_i_* as

$$\begin{array}{l} {\tilde{y}}_{i}=\{\begin{array}{ll} -1 & \;\;\text{if}\;y_{i}=0,\\ 1 & \;\;\text{otherwise}, \end{array}\\ \beta_{i}=\{\begin{array}{ll} 1 & \;\;\text{if}\;y_{i}\leq 1,\\ y_{i} & \;\;\text{otherwise}. \end{array} \end{array}$$

The RF and all CFs can now be solved for iteratively using *Algorithm 1*, where the choice of subproblem solver determines the nonlinearity (see *Eqs. 4*, *5*, and *6*). All three subproblems are convex, and unique solutions to these can, therefore, be found by using an appropriate solver. For the linear regression subproblem, this is particularly easy, as there is an analytical solution available:

$$w={\left(A^{⊺}A+\frac{1}{2C}\Gamma^{⊺}\Gamma \right)}^{-1}A^{⊺}(y-k),$$

where **A** is a matrix whose rows are given by $\nabla_{w}{\left(z_{i}\right)}^{⊺},i=1,\ldots ,N$, and **y** and **k** vectors containing the spike counts and constants for each sample *i*. When solving for the RF, this matrix is padded with a column of ones to also solve for the bias term. The logistic and Poisson subproblems, in contrast, need to be solved numerically. Here, we used the trust-region Newton method solver available in LIBLINEAR (Fan et al. 2008; Lee et al. 2015; Lin et al. 2008) with a tolerance of 1e-3. This solver utilizes both the gradient and the Hessian, which are given by

$$\nabla L_{\text{LogReg}}(w)=\Gamma^{⊺}\Gamma w+C\underset{i}{\sum }\beta_{i}[\sigma ({\overset{˜}{y}}_{i}z_{i})-1]{\overset{˜}{y}}_{i}\nabla_{w}(z_{i}),$$

$$\nabla^{2}L_{\text{LogReg}}(w)=\Gamma^{⊺}\Gamma +C\underset{i}{\sum }\beta_{i}\sigma ({\overset{˜}{y}}_{i}z_{i})[1-\sigma ({\overset{˜}{y}}_{i}z_{i})]\nabla_{w}(z_{i})\nabla_{w}{\left(z_{i}\right)}^{⊺},$$

$$\nabla L_{\text{PoiReg}}(w)=\Gamma^{⊺}\Gamma w+C\underset{i}{\sum }\left(e^{z_{i}}-y_{i}\right)\nabla_{w}(z_{i}),$$

$$\nabla^{2}L_{\text{PoiReg}}(w)=\Gamma^{⊺}\Gamma +C\underset{i}{\sum }e^{z_{i}}\nabla_{w}(z_{i})\nabla_{w}{\left(z_{i}\right)}^{⊺},$$

where σ(*yz*) is the standard logistic function ${\left(1+e^{-yz}\right)}^{-1}$. Note that even if these expressions are the same as for vanilla logistic and Poisson regression, off-the-shelf solvers are unlikely to do the job due to the constant *k_i_* in *Eq. 3*: these solvers normally have *z* predefined without this sample-specific constant.

### CF Origin

The CF needs a fixed origin for determining the local region that influences each RF element. The origin can be chosen freely and when no prior information is available on what type of contextual effects are present, the center element provides a conservative starting point. However, for RFs that include a time dimension (progressing left to right), it makes sense to fix the origin close to the right edge, as future events are unlikely to affect neural responses. As illustrated in Fig. 1*B*, we selected the origin three elements from the right edge of the CF under the assumption that responses, nonetheless, could be suppressed or enhanced by stimuli in the two following frames. However, separate tests confirmed that the specific location of the origin has little effect on our results. Furthermore, we fixed the CF parameter at the origin to zero so as to only include interaction terms in the CF.

### Regularization

In *Eqs. 4*, *5*, and *6*, we guard against overfitting by using a Tikhonov regularization term $||\Gamma w|{|}^{2}$ and a cross-validation step for selecting the regularization parameter *C*. However, running cross-validation on every iteration of *Algorithm 1* is computationally expensive. Therefore, we ran *Algorithm 1* to convergence with the regularization parameter set initially to *C* = 0.1, and then finalized the estimation with one additional iteration with five-fold cross-validation on the training data for both *steps 4* and *5*. Convergence was defined to have occurred when the relative decrease in the loss function between iterations was less than 0.01%.

We tested two different regularization matrices (Γ): one diagonal matrix and one representing the two-dimensional discrete Laplace operator. The first option, hence, corresponds to standard *l*^2^-norm regularization, whereas the second favors smooth solutions, similarly to the regularization used by Machens et al. (2004).

### Complementary Solutions

The overall optimization problem for estimating context models is nonconvex, even though the subproblems solved within *Algorithm 1* are convex. This means that *Algorithm 1* can converge to locally optimal solutions, and the fact that RF and CF parameters are multiplied with each other means that the signs of these parameters might be arbitrary. Indeed, we find that it is almost always possible to find complementary solutions where the signs in the RF and CFs are flipped. Therefore, for consistency, whenever a solution with a large negative RF value was found, we flipped the signs of all elements in the RF and reran *Algorithm 1* to completion.

### Naming Convention

We name the context models according to the subproblem solved. Thus, LinRegCtx, LogRegCtx, and PoiRegCtx refer to models with a nonlinearity that is the identity, the logistic, or the exponential function, respectively.

### Multifilter LN Models

The multifilter LN model, just as the context model, can be viewed as an extension of the traditional LN model, but instead of adding CFs, it includes additional linear filters. These filters, sometimes called features, are thought to span the subspace relevant for generating cellular responses (de Ruyter Van Steveninck and Bialek 1988; Pang et al. 2016; Schwartz et al. 2006; Sharpee 2013; Sharpee et al. 2004). Therefore, the traditional LN model corresponds to a multifilter LN model that spans a one-dimensional subspace using only one linear filter. Further, multifilter LN models differ from context models by assuming that the relevant subspace is low-dimensional instead of it being approximated using an RF-CF combination. We emphasize that the essence of multifilter LN models is the assumption that there is a low-dimensional stimulus subspace that drives the cell and that, therefore, the number of filters should be much lower than the dimensionality of the stimulus. Without this assumption, the division between multifilter LN and context models becomes redundant, as also context models can be described in the multifilter LN model form when enough filters are added. This restriction on the number of filters coincides with the restraint faced in practice when the amount of data is limited. Thus, the separation between multifilter LN and context models is both a fundamental one and a practical one. Subunit models (Vintch et al. 2012, 2015), which like the context models cross-correlate the stimulus with a filter, diverge from multifilter LN models in a similar fashion, and, thus, we have refrained from classifying these as multifilter LN models.

### Multifilter LN Model Estimation

Multifilter LN models have traditionally been estimated using spike-triggered analyses (Paninski 2003a; Rust et al. 2005; Touryan et al. 2002). Assuming a whitened and mean-centered stimulus, these models rely on the spike-triggered average (STA) and the spike-triggered covariance (STC) matrix (Pillow and Simoncelli 2006), defined as

$$\mu =\frac{1}{N_{\text{spikes}}}\underset{x_{i}|\text{spike}}{\sum }x_{i},$$

$$\Lambda =\frac{1}{N_{\text{spikes}}}\underset{x_{i}|\text{spike}}{\sum }(x_{i}-\mu ){\left(x_{i}-\mu \right)}^{⊺},$$

where μ denotes the STA, Λ is the STC matrix, *N*_spikes_ is the total number of spikes observed, and **x**_i_ | spike denotes the input vectors associated with spikes. In the most basic version, STC analysis, the linear filters of the multifilter LN model are extracted from the eigenvectors of the STC matrix (Touryan et al. 2002). Here, we refer to models estimated in this way as STC*x*, where *x* is an integer indicating the number of filters included. This simple analysis, however, may fail to take the STA into account, and it provides no quantification as to which of two filters is the more important if one is inhibitory (eigenvalue ≪1) and the other excitatory (eigenvalue ≫1). These problems are solved by, for example, the information-theoretic spike-triggered average and covariance (iSTAC) analysis (Pillow and Simoncelli 2006), which searches for a set of basis vectors (**B**)—the linear filters of the multifilter LN model—that span a subspace while maximizing the mutual information between spike arrival times and the spanned subspace. If one assumes that the stimulus distribution is Gaussian and white and that the spike-triggered ensemble is Gaussian, then the mutual information can be determined as

(7) $$I_{\text{iSTAC}}=\frac{\text{Tr}\left(B^{⊺}(\Lambda +\mu \mu^{⊺})B\right)-\log\left|B^{⊺}\Lambda B\right|-m}{2\log(2)},$$

where Tr() and || indicate the matrix trace and determinant, respectively, and *m* is the number of columns in **B**. Thus, the columns of **B** represent directions in space that affect spiking. We followed the approach in Pillow and Simoncelli (2006) to find **B**, and we refer to multifilter LN models estimated in this way by iSTAC*x*, where *x* is again an integer indicating the number of filters included. However, we note that our analytical expression for the gradient, used during the search for **B**, is different from the one provided by Pillow and Simoncelli (2006). We used

$$\nabla I_{\text{iSTAC}}(B)=\frac{\left(\Lambda +\mu \mu^{⊺}\right)B-\Lambda B{\left(B^{⊺}\Lambda B\right)}^{-1}}{\log(2)},$$

which we verified through gradient checking.

Both the traditional STC analysis and the iSTAC analysis have the advantage of being computationally lightweight, as they only deal with the subset of stimuli that elicited spikes. However, both are severely restricted by the assumption of Gaussian stimuli (but see Samengo and Gollisch 2013).

### Estimating the Nonlinearity

The final output of the multifilter LN model is obtained as a nonlinear transformation of the responses from all the linear filters. Once the filters are obtained, the nonlinearity can be derived by using Bayes rule as (Atencio et al. 2008; Chichilnisky 2001; Schwartz et al. 2006):

(8) $$f(z_{i})=P(\text{spike}|z_{i})=P(\text{spike})\frac{P(z_{i}|\text{spike})}{P(z_{i})},$$

where *P*(spike) was replaced with the average firing rate, and where $P(z_{i}|\text{spike})$ and $P(z_{i})$ are estimated using histograms. Unless otherwise mentioned, we used a resolution of 20 bins per dimension. This means that arriving at the nonlinearity requires the estimation of 20 additional parameters for traditional LN models, 400 additional parameters for two-filter LN models, and so on. The LN model hence runs into two problems when multiple filters are added: *1*) the number of parameters needed for approximating the nonlinearity grows exponentially ($20^{N_{\text{filters}}}$), and *2*) we can no longer visualize the nonlinearity when more than three filters are used (although it may in some cases be possible to simplify the nonlinearity Kaardal et al. 2013).

### Low-Rank QN Models

The low-rank QN model strives to side-step the problem of having to estimate and visualize a nonlinear mapping from a high-dimensional space, while still holding on to the concept of linear filters that span a relevant subspace (Fitzgerald et al. 2011; Kozlov and Gentner 2016; Park et al. 2013; Rajan et al. 2013). This is done by restricting the response to be a nonlinear function of the quadratic form of the stimulus, effectively letting the nonlinearity perform a mapping from a space of lower dimensionality than the subspace (Park and Pillow 2011; Sharpee 2013). Here, we implemented a refined version of the maximum noise entropy (MNE) method for QN model estimation by Fitzgerald et al. (2011). This method assumes that the nonlinearity is the logistic function, and it gives the similarity score as

(9) $$z_{i}=c+x_{i}^{⊺}v+x_{i}^{⊺}Jx_{i},$$

where the model parameters *c*, **v**, and **J** are selected so that the mean firing rate, as well as the first- and second-order correlations of the model predictions, match the observed ones. The final low-rank QN model filters are then obtained by diagonalizing *Eq. 9*, essentially selecting the eigenvectors of **J** that correspond either to the eigenvalues with the largest absolute values or, alternatively, to the vector **v** (see Fitzgerald et al. 2011 for details). As we are interested in creating models with a fixed number of filters (denoted by *N*_filters_), we followed the following procedure: *1*) select the eigenvectors that correspond to the *N*_filters_ −1 eigenvalues with the largest absolute values; *2*) project out the selected filters from **v**; *3*) select the last filter to be either **v** or the eigenvector with the *N*_filters_ th largest absolute eigenvalue, depending on which gives the model a higher mutual information value (defined below). The similarity score for the resulting low-rank QN model can then be described in one of the following two forms:

(10) $$z_{i}=x_{i}^{⊺}w_{v}+\overset{N_{\text{filters}}-1}{\underset{m}{\sum }}\lambda_{m}{\left(x_{i}^{⊺}w_{m,J}\right)}^{2},$$

$$z_{i}=\overset{N_{\text{filters}}}{\underset{m}{\sum }}\lambda_{m}{\left(x_{i}^{⊺}w_{m,J}\right)}^{2},$$

where **w** denotes filters of the low-rank QN model, λ is an eigenvalue, and the subscripts *v* and *J* indicate from which term in *Eq. 9* the filter originates.

Prediction-to-data matching of the mean firing rate, as well as of the first- and second-order correlations, is equivalent to maximizing the likelihood of the data (Fitzgerald et al. 2011; Malouf 2002). Therefore, we can find the parameters (*c*, **v**, and **J**) by solving a regularized logistic regression problem. This was again done by calling the LIBLINEAR solver, but this time by modifying the calculations for the gradient and the Hessian, so that all quadratic terms were recalculated on the fly, when needed. We did this to keep down the size of the data matrix (which would otherwise have grown to 70 GB). However, our resulting filters might still be suboptimal as the final expression for the similarity score (*Eq. 10*) differs from the one used during optimization (*Eq. 9*). Unfortunately, direct usage of *Eq. 10* leads to a nonconvex optimization problem, but we can, nonetheless, use gradient descent to refine all filters. For a logistic nonlinearity, the needed gradients are

$$\nabla L_{\text{MNE}}(w_{v})=\Gamma^{⊺}\Gamma w_{v}+C\underset{i}{\sum }\beta_{i}\left[\sigma ({\overset{˜}{y}}_{i}z_{i})-1\right]{\overset{˜}{y}}_{i}x_{i},$$

$$\nabla L_{\text{MNE}}(w_{m,J})=\Gamma^{⊺}\Gamma w_{m,J}+C\underset{i}{\sum }\beta_{i}\left[\sigma ({\overset{˜}{y}}_{i}z_{i})-1\right]{\overset{˜}{y}}_{i}2\lambda_{m}x_{i}^{⊺}w_{m,J}x_{i},$$

where we again included a Tikhonov regularization term with Γ being the identity matrix. The regularization parameter *C* was in the first LIBLINEAR part selected as the one with best performance on the training set (as this gave us filters with structure instead of noise) and in the second refinement part by evaluating model performance on a hold-out validation set not used for either training or testing. Selecting the regularization parameter based on training performance in the first part might contribute to overfitting, but separate experiments showed that the performance boost from the second refinement stage was robust against the exact regularization value used with LIBLINEAR, as long as the found filters had a non-noisy structure.

### Maximizing Mutual Information for Arbitrary Stimuli

Multifilter LN models and context models describe neural behavior in different ways, and therefore, it is possible that one type captures the behavior of real cells better than the other. A fair comparison is difficult to make unless models from both frameworks are optimized using the same measure. This can be achieved by, for example, maximizing the mutual information I between spike arrival times and the similarity scores (Adelman et al. 2003; Brenner et al. 2000; Fairhall et al. 2006, 2012; Sharpee et al. 2004), with the single-spike information defined as

(11) $$I=\int P(z|\text{spike})\log_{2}\left[\frac{P(z|\text{spike})}{P(z)}\right]dz,$$

where *P*() denotes probability distributions estimated with histograms. Each similarity score is weighted equally when estimating *P*(**z**), but according to the corresponding spike count (*y_i_*), when estimating *P*(**z** | spike), as suggested in Sharpee (2007).

Multifilter LN models that are estimated by maximizing *Eq. 11* are usually referred to as MID models, as these find filters that represent the most informative dimensions (MIDs) in stimulus space (Sharpee et al. 2004, 2006). Such models consequently have a close connection to iSTAC models as they both search for a maximally informative subspace. The MID model is the more general one, as *Eq. 11* reduces to *Eq. 7* when both distributions are Gaussian and when the stimulus distribution is whitened. Therefore, MID models have an advantage over iSTAC models, as well as over STC models, in that they make no assumptions about the stimulus distribution. However, maximizing I directly poses two challenges: *1*) *Eq. 11* is nonconvex and difficult to optimize, and *2*) it can only be used for estimating multifilter LN models with very few filters because the probability distributions are approximated with histograms (Williamson et al. 2015). The second problem is, hence, specific to the multifilter LN model, whereas the first is faced by context models and multifilter LN models alike.

Nonconvexity means, in principle, that a search would have to be done over the whole parameter space to find a global maximum. This is not feasible in practice, and an alternative is to use prior information to restrict the search, for example by specifying an initial guess of the optimal parameter values. This initial guess can then be improved upon by following a steepest ascent approach, where parameter values are updated by taking steps along the gradient. Expressions for the partial derivatives are, therefore, needed, and these have been provided for the multifilter LN model (Sharpee et al. 2004, 2006). Following this previous derivation, we note that the partial derivatives for the context model’s parameters are

$$\nabla I(w_{\text{rf}})=\int \frac{P(z)}{\log(2)}[<\nabla z(w_{\text{rf}})|z,\text{spike}>-<\nabla z(w_{\text{rf}})|z>]\left[\frac{d}{dz}\frac{P(z|\text{spike})}{P(z)}\right]dz,$$

$$\nabla I(w_{{\text{cf}}_{l}})=\int \frac{P(z)}{\log(2)}[<\nabla z(w_{{\text{cf}}_{\text{l}}})|z,\text{spike}>-<\nabla z(w_{{\text{cf}}_{\text{l}}})|z>]\left[\frac{d}{dz}\frac{P(z|\text{spike})}{P(z)}\right]dz,$$

where <∇z(w)|z> denotes the average gradient with similarity score *z*, and <∇z(w)|z,spike> denotes the average gradient with similarity score *z* that also led to a spike.

Here, we estimated MID versions of both multifilter LN and context models by maximizing I directly. In both cases, existing models were improved upon by additionally maximizing *Eq. 11* using gradient ascent. Regularization was obtained through the use of a separate validation set. The final model parameters were selected as those that obtained the highest I values on the validation set during the gradient ascent routine. The initial guesses were in all cases taken to be parameter values found using models presented in previous sections, for example, PoiRegCtx, STC, or iSTAC models. We refer henceforth to these models as MID*x*_init, where *x* denotes the number of filters for multifilter LN models and the descriptor “init” names the model that was used for generating the initial guess.

### Preprocessing

The original data were preprocessed using basis functions. We implemented both a dark and a bright basis function: The dark basis function resulted in binary vectors, where a dark bar was coded with unity and a bright bar was coded with zero, whereas the situation was reversed for the bright basis function. All traditional and multifilter LN models were estimated using the bright basis function only, while context models were estimated using two types of input vectors: *1*) the bright basis function only and *2*) a concatenated vector consisting of both the dark and the bright basis function. The latter created an input that can be thought of as a space-time-intensity pattern. Additionally, we estimated the context models using the original input values (−1 and 1) as a check for potential mean-shifting and scaling effects, but no such effects were found.

Note that the concatenated bright-dark input vector is of no use for traditional LN models, as the bright and dark basis functions are linearly dependent. Context models, in contrast and perhaps counterintuitively, can benefit from the dark basis. These models solve a constrained optimization problem where the constraints derive from multiple RF elements sharing the same CF. It is easy to show by example that including a dark basis function can effectively relax these constraints by increasing the number of parameters available for expressing the similarity score. This, in turn, allows the model to express mappings that would be impossible if only the bright basis function were used.

### Model Evaluation and Finite Sample Bias

Model performance was primarily measured as the mutual information between spike arrival times and the similarity score **z**. However, mutual information as calculated in *Eq. 11* suffers from a finite sample effect, as the probability distributions are estimated using histograms (Paninski 2003b; Panzeri and Treves 1996; Treves and Panzeri 1995). We corrected for this bias by using a procedure introduced by Fairhall et al. (2006), where the bias is estimated using I values calculated from null features (obtained from STC analysis). Such null features should have I values of zero, and obtained values are, hence, estimates of the bias. However, the bias is also affected by the effective number of bins that are being used (Panzeri and Treves 1996), that is, by how many of all available bins that actually get nonzero values (certain *z*_1_ and *z*_2_ combinations might never occur when estimating a two-dimensional distribution). A higher number indicates that each value is estimated from fewer samples, and this causes a larger bias. Therefore, we used different histogram resolutions to estimate the bias from the null features as a function of the average number of nonzero bins in *P*(*z*) and *P*(*z* | spike). Our correction procedure was then the following: *1*) calculate a naive I value using *Eq. 11* and determine the average number of nonzero bins, *2*) interpolate the sampled bias function to find the expected bias for the observed number of nonzero bins, and *3*) subtract the expected bias value from the naive value to get the bias-corrected I value. By following this procedure, we were able to calculate bias-corrected I values for different histogram resolutions. This, in turn, allowed us to verify the success of the bias correction, because the true value of I should be independent of the resolution used (assuming that the resolution can sufficiently approximate the probability distribution).

**Fig. 2.**
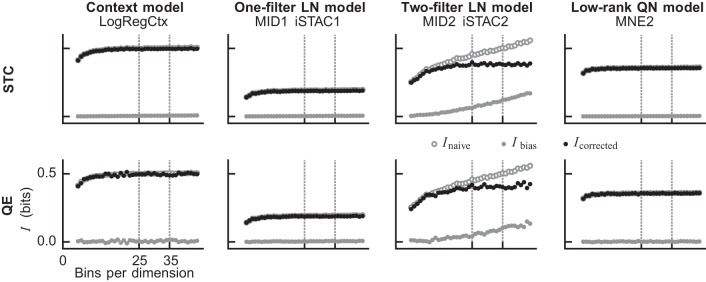
Estimation of the finite sample effect. The two bias correction methods spike-triggered covariance (STC) and quadratic estimation (QE) resulted in near-identical bias estimations. For models that only need to approximate one-dimensional probability distributions, such as context models, low-rank quadratic-nonlinear (QN) models, and traditional linear-nonlinear (LN) models, the bias is negligible. Thus, the bias-corrected I value (black), obtained by subtracting the bias (filled gray) from the naive value (open gray), is essentially the same as the naive value, which it overlays (as a result, the naive values are hidden beneath the bias-corrected values). This is in contrast to models that need to estimate two-dimensional probability distributions (general two-filter LN models), where the bias increases steadily as a function of the histogram resolution. Thus, the initial rapid increase for low bin numbers stems mainly from information loss caused by the resolution being too coarse, whereas the later gentler increase is due to the finite sample effect (see Treves and Panzeri, 1995; Adelman et al., 2003).

We also implemented a second step for verifying our bias correction by comparing the null-feature correction method to the more commonly used quadratic estimation (QE) method (Adelman et al. 2003; Atencio et al. 2008, 2012; Panzeri et al. 2007; Strong et al. 1998). The QE method is computationally more demanding, as it estimates naive I values from subsampled fractions of the data and uses quadratic estimation to infer what the correct I value would be for infinitely many samples. Figure 2 compares the results obtained with the two methods for one-dimensional probability distributions (context models, one-filter LN models, and low-rank QN models), and two-dimensional distributions (general two-filter LN models). The naive value, the estimated bias, and the bias-corrected values are all presented as a function of the number of bins used per dimension when estimating the histograms. Three observations stand out: *1*) too few bins lead to information loss; *2*) the bias can be very large for two-dimensional probability distributions; and *3*) the two methods find very similar bias estimates, but the estimate given by the QE method fluctuates more. Importantly, both methods result in a bias-corrected value that fluctuates around a fixed value (ignoring the initial region with information loss). Therefore, we took our final estimate to be the mean of the bias-corrected values obtained for the resolutions 25, 26,…, 35 bins per dimension using the STC method. In some cases, we observed that the bias-corrected value retained a positive slope for the general two-filter LN models, indicating that the bias was probably underestimated. This was especially apparent on the testing sets that had fewer samples to start with. Therefore, it is likely that, for some cells, the general two-filter LN models have a somewhat overestimated I value on the testing sets.

Another popular method for evaluating model performance is the correlation coefficient (*r*) between model predictions and real responses (Chen et al. 2007; Touryan et al. 2005; Vintch et al. 2015). For repeated stimuli, this method can be further extended to estimating the explainable variance captured by the different models (Sahani and Linden 2003; Schoppe et al. 2016). For current purposes, however, the correlation coefficient has two drawbacks: *1*) the data set we used does not include stimulus repetitions, and *2*) the correlation coefficient requires access to model predictions. The latter has the consequence that additional model parameters are needed for estimating the nonlinearities (roughly doubling the total number of parameters for a two-filter LN model), and we observed that this tended to cause overfitting. The general two-filter LN models obtained very high *r* values on training data but not on test data, and we found it difficult to know whether the difference to context models was real or just caused by overfitting. Mutual information differs in this regard as it does not depend on the number of parameters used for estimating the nonlinearity (assuming that the finite sample effect is corrected for). Therefore, it lets us compare all of the models without having to worry about potential overfitting when estimating the nonlinearity. We have, nonetheless, included the correlation coefficient as a secondary measure in our analysis. This is useful both as a control and as a way of dealing with general two-filter LN models whenever data are scarce. We observed that the mutual information tended to be overestimated in these cases (due to finite sample effects), whereas the correlation coefficients tended to be underestimated (due to overfitting).

### Training and Testing Sets

We used a five-fold training/testing paradigm where the available data for each cell was split into five folds. Models and potential hyperparameters (*C*) were estimated by dividing the data from four folds into training (80%) and validation (20%) sets, whereas data from the fifth fold was used for testing only. Hence, we trained and evaluated five versions of the same model for each cell, using different folds for training and testing. The evaluation scores presented throughout this article are, therefore, mean values over five trials.

## RESULTS

The traditional single-filter LN model can be extended in accordance with various model frameworks that include the traditional LN model as a special case. Here, we compared a range of models from the context- and multifilter LN-model frameworks to examine which of these frameworks is best at capturing the behavior of complex cells. The experimental data were obtained from the CRCNS program (Dan et al. 2009), and it consisted of recorded stimulus-response data from 61 complex cells in cat striate cortex (detailed description in Touryan et al. 2002). These cells were stimulated using a sequence of pseudo-random bar patterns, and we created input vectors (**x***_i_*) from the stimuli by concatenating the bar patterns from the previous 16 frames. Each input vector, thus, contained the pseudo-random bar patterns presented during the past 267 ms (60-Hz presentation rate), and it may be viewed as a 16 × 16 space-time pattern or as a direction in the 256-dimensional stimulus space. Responses (*y_i_*), in turn, denoted the number of spikes that occurred during the presentation time of each frame.

All of the models evaluated in this study computed a (possibly multidimensional) similarity score (**z***_i_*) for each input pattern and mapped this score to a predicted firing rate through a nonlinearity (*f*), as detailed in materials and methods. Quantitatively, each model’s performance was evaluated primarily using the mutual information (I) between spike events and the similarity score, and secondarily using the correlation coefficient (*r*) between predicted and recorded responses. Additionally, the models were further qualitatively evaluated on the basis of their ability to provide a description of each cell’s stimulus-response mapping. Model complexity was varied by estimating context models with one or two CFs and multifilter LN models with two or four filters. This allowed us to compare models of varying complexity while ensuring that in each comparison, the models had the same number of parameters.

**Fig. 3.**
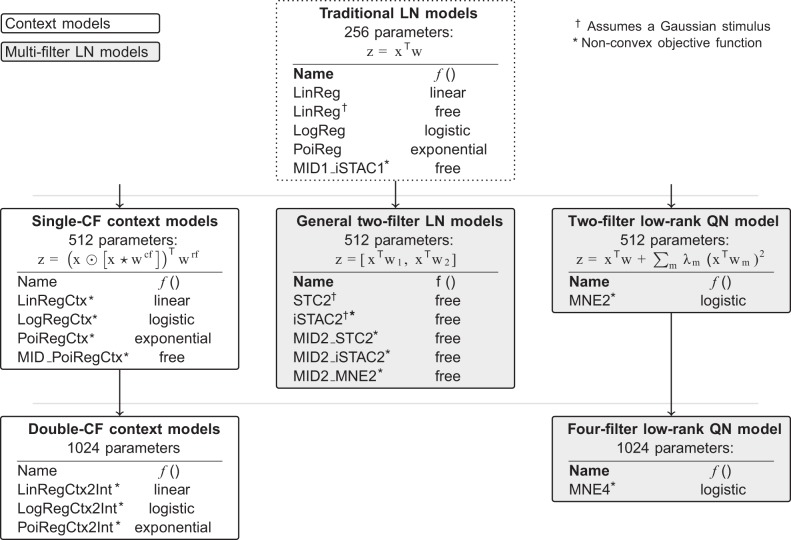
Overview of the estimated models. These are characterized by the number of parameters (excluding a potential bias term), the similarity score (⊙ and ⋆ denote element-wise multiplication and cross-correlation, respectively), the nonlinearity (“free” meaning no assumptions), and the assumptions about the stimuli. Extending the traditional linear-nonlinear (LN) model with one filter (the receptive field, RF), the more complex models can be classified as being either context models or multifilter LN models. Our initial baseline set-up compares context models using a single context field (CF) two-filter LN models, and traditional LN models with one another. Thereafter, we compare the performance of double-CF context models with that of a four-filter LN model (MNE4).

We begin by simulating a complex cell and looking at how well single-CF context models and two-filter LN models describe the stimulus-response mappings of such a cell. Thereafter, we look at how well the models perform on real experimental data. As overviewed in Fig. 3, this presentation is organized according to model complexity: from a baseline set-up to more complex models. The baseline set-up consists of four types of traditional LN models (LinReg, LogReg, PoiReg, MID1_iSTAC1), six two-filter LN models (MNE2, STC2, iSTAC2, MID2_MNE2, MID2_STC2, MID2_iSTAC2), and four single-CF context models (LinRegCtx, LogRegCtx, PoiRegCtx, and MID_PoiRegCtx), where the underscore in all cases denotes initial solutions. The more complex models, in turn, comprise a four-filter LN model (MNE4) and three space-time-intensity context models with two CFs (LinRegCtx2Int, LogRegCtx2Int, and PoiRegCtx2Int). Two of the multifilter LN models (MNE2 and MNE4) were estimated as a specific type of low-rank QN model, where the model’s nonlinearity is constrained, so that it only needs to perform a mapping from one-dimensional space. This lets one avoid the curse of dimensionality, which is normally encountered when estimating the nonlinearity for general multifilter LN models.

All context models (except MID_PoiRegCtx) were estimated using either standard *l*_2_ norm regularization or smooth regularization. As we observed no clear differences between the two regularization methods, the results below were all obtained by using smooth regularization.

### Simulated Feedforward Complex Cell

**Fig. 4.**
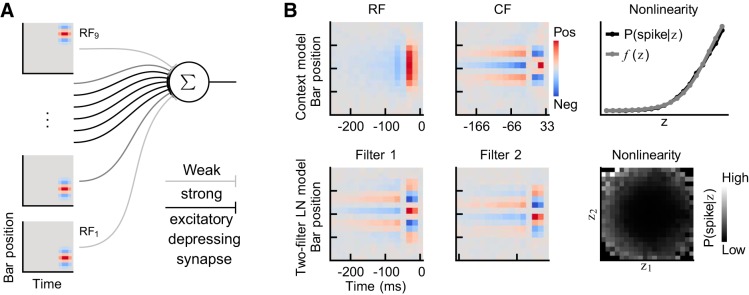
Comparisons of context- and multifilter linear-nonlinear (LN) models with simulated data. *A*: nine input cells with spatially shifted linear receptive fields (RFs) drove a complex output cell through excitatory depressing synapses. Each cell was of the leaky integrate-and-fire type, and each input cell was stimulated by a pseudo-random binary bar stimulus. *B*: the two models describe the simulated complex cell in very different ways. The context model describes the complex cell’s responsive region in the RF and captures the spatially shifted linear RFs of the input cells in the rightmost part of its context field (CF). In contrast, the two-filter LN model finds two 90-degree phase-shifted filters that constitute a linear combination of multiple spatially shifted input cell RFs. This linear combination further requires that the two-filter LN model have a symmetrical nonlinearity, whereas the context model should have a monotonically increasing nonlinearity. Both models, nonetheless, depict contextual effects brought about by synaptic depression—these are evident in the CF and the filters as opposite-sign trailing regions in time.

We utilized the modeling setup from our previous study (Westö and May 2016) and generated simulated data from a small network depicted in Fig. 4*A*. This network consisted of an output cell driven by nine input cells with identical but spatially shifted linear RFs and, thus, realized the hierarchical description of a complex cell (Martinez and Alonso 2003). All 10 cells were of the leaky integrate-and-fire type, and input to the network consisted of binary pseudo-random bar stimuli. Each input cell, in turn, drove the output cell via an excitatory depressing synapse (*u* = 0.9 and $\tau_{\text{rec}}=200$ ms, see Tsodyks and Markram 1997). For further details on the computational modeling methods, see Westö and May (2016).

All models within the same framework resulted in almost identical descriptions of the simulated complex cell. However, as illustrated in Fig. 4*B*, the difference between the frameworks is clearly observable. The context model, which first cross-correlates the stimulus with the CF and subsequently scales each element in **x***_i_* with the result, provides a very different quantization of neural behavior than the multifilter LN model. In fact, the operation of the context model results in a quantization where the region in space and time that can affect spiking behavior is indicated in the RF and where the CF gives the stimulus context needed to activate the RF. For the complex output cell, the RF, hence, enhances the region in space and time covered by all input cells, whereas the CF captures both the linear RF of the input cells, as well as the effects of synaptic depression. The spatially shifted RF of the input cells is found in the rightmost part of the CF, while the CF’s negative tail, following the positive region in time, is representative of synaptic depression. The negative tail provides a context in which each input cell is only likely to drive the output cell if the input cell has recently remained inactive. Thus, the tail describes the contextual effects arising from synaptic depression.

Looking at the two-filter LN model next, we note that *1*) its filters appear to be phase-shifted 90° (a quadrature pair; Adelson and Bergen 1985) and *2*) the filters seem to be constructed as linear combinations of multiple spatially shifted versions of the linear RF of the input cells. This is no coincidence: the two-filter LN model does not include a specific RF part (as in the case of the context model) and, consequently, it must show sensitivity to the RF pattern over a wider region by including multiple spatially shifted versions of this pattern in its filters. For example, subindexing the RFs of the input cells as in Fig. 4*A*, the first filter appears to be equivalent to RF_3_ − RF_5_ + RF_7_, whereas the second filter corresponds to RF_4_ − RF_6_. This inclusion of negative weighting factors requires that the nonlinearity of the two-filter LN model is symmetrical, as a stimulus corresponding to, for example, RF_5_ will result in a strongly negative similarity score. We note that, besides the alternating pattern along the spatial dimension, the filters include trailing regions of opposite signs caused by synaptic depression, just as in the context model’s CF.

We conclude that the context model provides a more accurate and descriptive quantization of the simulated complex cell. The rightmost part of its CF accurately captures the input cells’ spatially shifted RF, and its RF correctly depicts the region in space and time that elicits spikes. In contrast, the two-filter LN model finds filters that do not represent any of the network’s fundamental building blocks as such, but rather, are equivalent to different linear combinations of these building blocks. Consequently, it is not obvious from the two-filter LN model what the RF of the input cells looks like, nor is it obvious that the trailing regions derive from synaptic depression. These qualitative observations on the superiority of the context model are paralleled by quantitative measures of performance, whereby the context and two-filter LN model reach I values of 0.93 bits and 0.63 bits, respectively. The context model, thus, seems to include stimulus-response mappings that better approximate a pooling operation over input cells with identical but shifted RFs (i.e., a hierarchical description of a complex cell). To verify that this conclusion is not due to biased mutual information calculations, we simulated additional data using only two input cells (RF_4_ and RF_6_) and no synaptic depression. This approximates a situation in which the output cell’s behavior is characterized by a two-dimensional stimulus subspace, and, thus, reflects a situation where the multifilter LN model should perform better. Indeed, the two-filter LN model outperformed the context model on data from this simplified network (I values of 2.00 bits and 1.67 bits, respectively). On the basis of these observations, we would expect that context models only outperform two-filter LN models in those cases in which they truly provide a better description of the data.

### One CF Vs. Two Filters

**Fig. 5.**
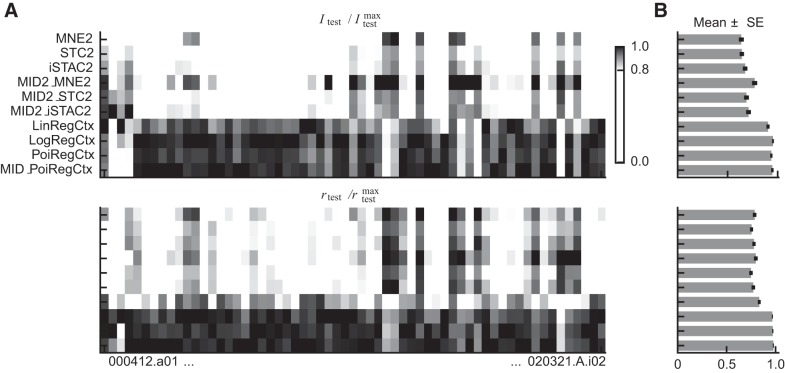
Normalized mutual information values ($I/I_{\text{max}}$) and correlation coefficients (*r*/*r*_max_) for each cell separately. *A*: the normalized values on the testing sets for each cell reveals that the context models performed the best for 46 out of 61 cells (75.4%) using the mutual information measure, and for 51 out of 61 cells (83.6%) using the correlation coefficient. *B*: the normalized values averaged over all cells summarize our conclusion that context models perform clearly better than equally complex (same number of parameters) two-filter linear-nonlinear (LN) models. Error bars denote means ± SE.

Our baseline results for the data of Touryan et al. (2002) are summarized in Fig. 5, which shows the normalized $I\;/\;I^{\text{max}}$ and *r*/*r*^max^ values for each of the baseline models. $I^{\text{max}}$ and *r*^max^ were determined separately for each cell, and they denote the values obtained by the best-performing model. The values are, hence, normalized cell-specific measures of how well a particular model compared against the best model for that cell. The normalized values are presented for each cell separately in Fig. 5*A* and as an average over all cells in Fig. 5*B*. In general, the two normalized values paint a similar picture, and therefore, below, we will mainly focus on the I values, as these were our primary performance measure.

Focusing on the two-filter LN models first, we note that the MNE2, STC2, and iSTAC2 models were the ones with the worst performance (mean I ± SE on the testing sets: 0.64 ± 0.02, 0.64 ± 0.02, and 0.68 ± 0.02, respectively). This outcome was to be expected: the two-filter low-rank QN model is a restricted two-filter LN model and should, in general, perform worse than the general two-filter LN models. Moreover, both the STC2 and the iSTAC2 models incorrectly assume a Gaussian stimulus distribution, and one would, therefore, expect them to underperform. This suboptimal performance can be observed in Fig. 5*A*, where the MNE2 model actually outperforms the STC2 and iSTAC2 models on a few cells. The general MID2 two-filter LN models make no assumptions about the stimulus distribution, and consequently, these are the best performing two-filter LN models (but only marginally and only with the I values). Interestingly, the MNE2 model appeared to provide the best initialization, as the MID2_MNE2 model was the two-filter LN model with the best overall performance (0.77 ± 0.03, 0.69 ± 0.02, and 0.71 ± 0.02 for MID2_MNE2, MID2_STC2, and MID2_iSTAC2, respectively).

Among the context models, the LogRegCtx and PoiRegCtx models performed almost identically (0.95 ± 0.01, 0.94 ± 0.01, respectively), whereas the LinRegCtx model performed slightly worse (0.91 ± 0.01, the LinRegCtx model’s relatively lower *r* values stem from using a linear *f*, instead of estimating it from data). A more realistic assumption about the nonlinearity, such as an exponential or a logistic function, therefore, appears to be advantageous compared with the linear relationship assumed by the LinRegCtx model. Like the two-filter LN models, the context models showed little signs of improvement when I was maximized directly using gradient ascent (MID_PoiRegCtx). Thus, it seems that the original context models already lie close to the local optima of the mutual information function. Nonetheless, this comparison was only done for the PoiRegCtx model.

Comparing the context models to the two-filter LN models using the normalized I values reveals that 46 out of 61 cells (75.4%) are better described using context models, whereas the remaining 15 cells (24.6%) are better described using two-filter LN models. That is, for 46 cells, one could find a single-filter context model that reached a higher I value than any of the two-filter LN models, and vice versa for the remaining 15 cells. Therefore, while context models fared better in most cases, neither model framework can be said to be universally the best. This conclusion still holds if the mutual information score is exchanged for the correlation coefficient. In that case, the context models are superior for 51 out of 61 cells (83.6%). Out of the remaining 10 cells, eight belong to the group of 15 cells for which the two-filter LN models also had the highest I values. Interestingly, six out of these eight cells had absolute correlation coefficients that were among the highest observed (all in the top 8). Thus, the multifilter LN models tended to perform the best for those cells whose responses also could be predicted the best.

Previous studies have found that multifilter LN models are clearly superior to traditional LN models in describing the behavior of complex cells (Rust et al. 2005; Touryan et al. 2002). This superiority over traditional LN models was also observed here but for both context and two-filter LN models. The traditional LinReg, LogReg, and PoiReg models, all with monotonically increasing nonlinearities, achieved normalized I values in the 0.25–0.26 range, while the MID1 model with its unconstrained nonlinearity fared slightly better (0.38 ± 0.02). These scores are, nonetheless, clearly lower than those obtained by either context or two-filter LN models.

### Two Views and Two Interpretations

**Fig. 6.**
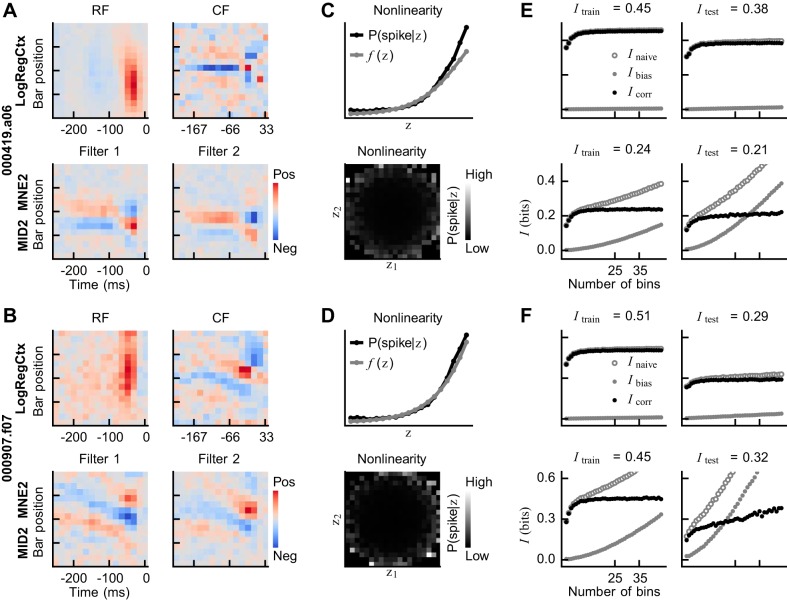
Model estimation and evaluation results for two representative cells (top vs. bottom). *A*: the context model’s receptive field (RF) and context field (CF) as well as the two-filter linear-nonlinear (LN) model’s filters for the first cell. The pattern to which the cell is sensitive is encoded in the CF of the context model, and the insensitivity to a spatial shift is seen in the elongated RF. In the case of the two-filter LN model, the pattern of the CF is present in both filters, and the insensitivity to a spatial shift emerges out of the spatial shift in the filters. *B*: the fields of the context model and the filters of the LN model for the second cell are shown as in *A*. The tilt in the CF and in the filters indicates that the cell is sensitive to a bar in motion. *C* and *D*: for both cells, the context model results in a monotonically increasing nonlinearity, whereas the two-filter LN model finds a symmetrical nonlinearity. *E*: in the case of the first cell, the context model outperforms the two-filter LN model in terms of the mutual information measure, as was the case for the majority of the cells. *F*: in contrast, the second cell illustrates an example where the two-filter LN model performed better on the testing sets, but where the bias is likely to be underestimated for the two-filter LN model [due to there being few spikes for estimating P(z|spike)].

We next illustrate how the estimated models represent real cell behaviors and see how this affects our understanding of how a cell’s preferred stimulus should be interpreted. This includes visualizing the fields/filters, the nonlinearity, and the bias-corrected I values. Figure 6 depicts the model estimation and evaluation results for two cells. We restrict our presentation to the LogRegCtx and MID2_MNE2 models, as all the context models found similar fields, and all of the two-filter LN models found similar filters. We also note that these two cells are representative of the entire ensemble of cells in terms of the estimated fields and filters.

Starting with the context model, we see that the CFs promote a dark-bright-dark pattern and that this pattern can be shifted along the spatial dimension due to the elongated RFs. We also note that the CF for the cell in Fig. 6*A* has a negative tail that is spatially narrower than the spatial width of the RF. This is a pattern that was present in roughly half of all cells; for 29% of all cells, it was only one bar wide (criterion: mean tail value across time at least four times larger than for neighboring bar positions). Such tails indicate that the cell can be facilitated only by dark-bright-dark patterns that have not recently been encountered. Additionally, roughly 10% of the cells had CFs with spatio-temporally tilted patterns, as in Fig. 6*B*, indicating sensitivity to a bar in motion.

Turning to the two-filter LN model next, we see that its filters contain patterns that are quite similar to the ones found in the CFs. As in our simulated network, the filters are phase-shifted by 90° and positioned so as to cover the whole region in the context models’ elongated RFs. Further resembling our simulations, the two-filter LN models obtain symmetrical nonlinear functions (Fig. 6, *C* and *D*), suggesting that the cells should respond both to the patterns in the two filters and to the inverse of these patterns.

In the previous section, we saw that for 75.4% of the cells, the context models reached higher I values than the two-filter LN models. Such a case is represented by the first cell in Fig. 6, where the performance measures for both the training and testing sets are shown in Fig. 6*E*. In contrast, the two-filter LN models performed better for the second cell, as shown in Fig. 6*F*. The I value used for the comparisons in the previous section is stated above each graph and corresponds to the mean bias-corrected value (i.e., the average over values gained with resolutions 25 to 35 bins per dimension). We note that when context models are used, the bias is small and, hence, the bias-corrected I values are stable across bin numbers for both the training and the testing sets. However, the I values of the two-filter LN model, requiring the estimation of a two-dimensional distribution, have a large bias that is not always correctly estimated for the testing sets. For some cells, for example, the one whose I values are shown in Fig. 6*F*, the bias-corrected I values retain an increasing trend, which probably indicates that the bias is underestimated. Thus, we suspect that the two-filter LN models’ I values on the testing sets might be overestimated for some cells. This implies that the difference in performance between the context and two-filter LN models could be even larger than what is shown here (a conclusion that is supported by evaluations using correlation coefficients instead of mutual information).

Finally, we underline that the I values in Fig. 6, *E* and *F* are themselves mean values over five separate training and testing configurations. While the model that was best in this mean sense was also the best in the individual configurations, the I values could vary quite heavily across the configurations. Hence, the models were in many cases better at describing certain parts of the whole data set.

### Beyond One CF and Two LN Filters

We suspected that the context models could do even better with more than one CF. To test this, we added an intensity dimension to the inputs by preprocessing the original input with dark and bright basis functions. The dark basis function encoded dark bars with zero and bright bars with unity, whereas the bright basis function did the reverse. This preprocessing, thus, gave the context models a space-time-intensity RF where sensitivity to dark and bright bars could be marked out separately. We estimated these context models (referred to as LinRegCtx2Int, LogRegCtx2Int, and PoiRegCtx2Int models) by running *Algorithm 1* to completion with one shared CF initially, and then again with two CFs: one for dark and the other for bright intensities. The second run, thus, used the parameters found from the first run as an initial guess when estimating the double-CF context models.

With their space-time-intensity RF and two CFs, these context models have as many parameters as a multifilter LN model with four filters. We do not, however, have enough data to estimate a nonlinear mapping from a four-dimensional space, nor can we visualize it. Therefore, we based our comparison between the context- and multifilter LN-model frameworks on a specific type of four-filter low-rank QN model, namely the MNE4 model. This model can be viewed as a four-filter LN model with additional constraints on the nonlinearity that in this case restricts it to a mapping from a one-dimensional space.

**Fig. 7.**
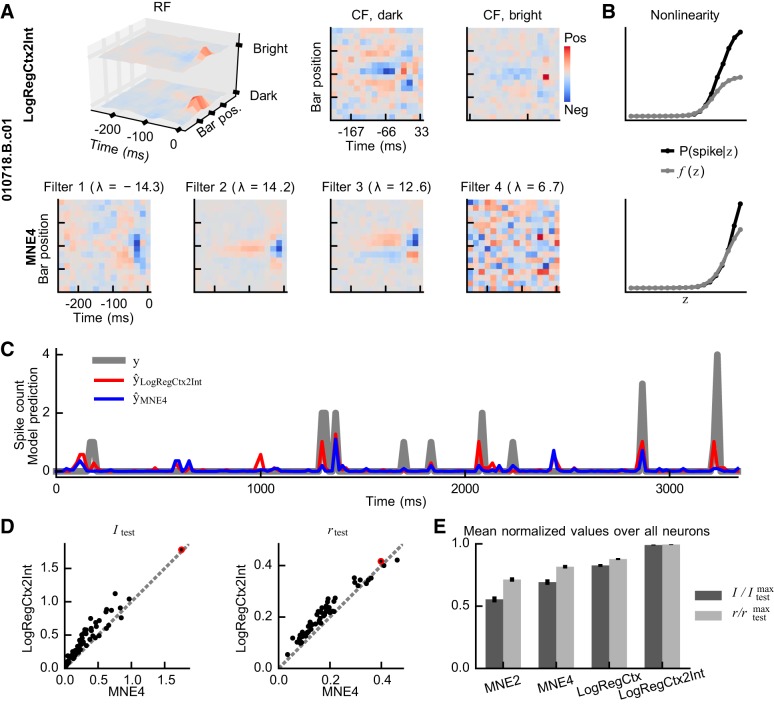
Context models with two context fields (CFs) vs. four-filter low-rank quadratic-nonlinear (QN) models. *A*: the patterns observed in the LogRegCtx2Int model’s CFs generally resemble the patterns seen in two of the maximum noise entropy (MNE4) model’s four filters, whereas the MNE4 model’s remaining filters contained noise or unrelated patterns. *B*: both models, nonetheless, associated higher similarity scores with a higher spike probability. *C*: however, their predicted responses could differ clearly from one another. The panel shows both the actual response from the test set, as well as predicted responses from the two frameworks (obtained using *Eq. 8* in both cases). *D*: the LogRegCtx2Int model, as well as its baseline version (LogRegCtx), were superior overall. These obtained I values that were on average 55% and 31% higher, respectively, than those obtained by the MNE4 model, and they also obtained higher *r* values. *E*: the normalized values averaged over all cells further highlight the performance difference between the two frameworks and illustrate that the context models performed the best. The red circle in *D* denotes the cell shown in *A–C*. The error bars denote means ± SE.

As with the baseline models, we observed that all context models found similar solutions and performed almost identically. Therefore, we only present results obtained with the LogRegCtx2Int model. Thus, Fig. 7*A* illustrates the RF and the CFs for the LogRegCtx2Int model, as well as the filters of the MNE4 model for one example cell. We note that the LogRegCtx2Int model contains two similar CFs whose pattern resembles both the single CF of the LogRegCtx model (not shown) and the pattern seen in two of the MNE4 model’s filters: all include a positive or negative region that is followed in time and flanked in the spatial dimension on at least one side by a region of opposite sign. This was the general outcome observed throughout the data set. That is, the two CFs in the double-CF model tended to resemble the CF of the single-CF model, as well as two of the filters in the MNE4 model. The other MNE4-model filters, in contrast, included noise or unique patterns, as in Fig. 7*A*. Both the LogRegCtx2Int and the MNE4 models, nonetheless, associated higher similarity scores with a higher spike probability, as evident by the monotonically increasing shape of *P*(spike | *z*) in Fig. 7*B*. The predicted responses could, however, differ clearly between the model frameworks, even in cases in which the performance of the models was found to be nearly identical. This is illustrated in Fig. 7*C*, where the predicted responses to part of the test set is shown for the same cell as in *A*. Overall, the LogRegCtx2Int model and its baseline model (LogRegCtx) were superior in terms of I values, which were on average 55% and 31% higher, respectively, than those obtained by the MNE4 model (the difference was similar but smaller for *r* values, see Fig. 7*C*). This performance difference is unlikely to be caused by having multifilter LN models with too few filters, as Touryan et al. (2002) observed that 50 out of the 61 cells in the data set only had two significant filters and that only one had more than four. Rather, the difference probably reflects a lack of sufficient data for estimating the required number of filters. That is, the multifilter LN model framework simply describes less useful stimulus-response mappings with its significant filters than the context model framework. A summary of these findings is given in Fig. 7*D*, where the normalized I and *r* values averaged over all cells are depicted for each model (I±SE: 0.55 ± 0.02, 0.63 ± 0.02, 0.82 ± 0.01, and 0.99 ± 0.00 for MNE2, MNE4, LogRegCtx, and LogRegCtx2Int, respectively).

Finally, the fields/filters in Fig. 7*A* highlight a problem with more complex models. When these are compared with their baseline versions, it is not easy to decipher what the added complexity corresponds to in terms of neural behavior. That is, it is very difficult to infer something new when looking at the fields of the double-CF model or when trying to mentally add up the contributions from each of the four filters in the MNE4 model. Therefore, we conclude that while these more complex models do reach higher I values, they fail to present this added information in an interpretable manner.

## DISCUSSION

RF models are commonly used for capturing, interpreting, and predicting neural behavior (Meyer et al. 2017; Ringach 2004; Sharpee 2013; Spillmann 2014; Spillmann et al. 2015). They make simplifying assumptions about stimulus-response mappings, allowing one to fit a restricted number of model parameters, according to stimulus-response data. Different models, however, make different simplifying assumptions, and this affects which stimulus-response mappings can be captured and how these are visually represented. The traditional LN model, with a single filter and an easily visualized nonlinearity, is easy to interpret but often too simplistic and in need of extension, as in the case of describing complex cells in visual areas (Chen et al. 2007; Pillow and Simoncelli 2006; Rust et al. 2005; Touryan et al. 2002). Here, we compared two commonly used frameworks for extending the traditional LN model: that of context models (Ahrens et al. 2008; Westö and May 2016; Williamson et al. 2016) and that of multifilter LN models (de Ruyter Van Steveninck and Bialek 1988; Samengo and Gollisch 2013; Schwartz et al. 2006), including low-rank QN models (Fitzgerald et al. 2011; Park et al. 2013; Rajan et al. 2013). Both include framework-specific stimulus-response mappings, and they also present these mappings in different ways. However, to our knowledge, these model frameworks have not been compared in terms of their performance. Therefore, this study represents the first attempt at such a comparison.

We fitted models of equal complexity (i.e., with the same number of parameters) from both frameworks to the spiking activity of 61 cells in cat striate cortex responding to temporal sequences of random bar stimuli (Touryan et al. 2002). Also, we developed novel ways to estimate context models with a more realistic (i.e., logistic or exponential) nonlinearity. Our first baseline comparison, between single-CF context models and two-filter LN models, showed that the mutual information between similarity scores and spike events was on average highest for the context models. Similarly, the context models were also the best on average at predicting responses to unseen stimuli, as measured by correlation coefficients. Taken together, this indicates that the simplifying assumption behind the context model framework matches the behavior of complex cells better than the assumption made by the multifilter LN model framework. Thus, the idea that cellular behavior is mainly affected by stimulus contexts appears more valid than the assumption of a low-dimensional relevant stimulus subspace. Furthermore, our new methods for estimating context models with logistic and exponential nonlinearities produced the best results.

In our second comparison, we examined the effects of adding filters or CFs to the models. General multifilter LN models scale badly in this regard because of the curse of dimensionality when estimating the nonlinearity. Therefore, we performed our comparison between double-CF context models and an additionally restricted multi filter LN model, that is, a four-filter low-rank QN model. This comparison showed that the double-CF context models were superior, obtaining mean I values that were 55% higher than those obtained by the low-rank QN model. Interestingly, the single-CF context model also outperformed the four-filter LN model (31% higher I values on average) despite having only half of the number of parameters. Touryan et al. (2002) originally observed that only one (1.6%) of the cells in the data set had more than four significant filters. Thus, the conclusion that the context model framework is superior for describing complex cells appears to hold even when all significant LN model filters are included. These results support the prediction of our previous modeling study (Westö and May 2016), whereby context models should perform better than multifilter LN models whenever input cells with identical but spatially/spectrally shifted RFs drive an output cell.

### The Case for Different Frameworks

Even though RF models are intended to provide interpretable quantizations of neural behavior, interpretability often suffers as models are made more complex. For example, the multifilter LN-model framework becomes increasingly difficult to interpret as more filters are added: the nonlinearity can no longer be visualized when more than three filters are used, and it becomes more and more difficult to mentally add up the effects of different filters as they increase in number. This means that unless the behavior of cells can be described with very few filters then, arguably, we might be using the wrong approach (but see Kaardal et al. 2013). An alternative approach is to use another model framework, such as the context-model one, with different underlying assumptions. Of course, context models will also become uninterpretable as more and more CFs are added. However, as demonstrated by our results, context models with only one CF can describe mappings that multifilter LN models can only achieve with three or more filters, and in these cases, context models are clearly superior.

We found that the context models performed better than equally complex two-filter LN models for 75.4% and 83.6% of the complex cells, measured by the mutual information and correlation coefficient, respectively. Similarly, others have found that subunit models (Eickenberg et al. 2012; Vintch et al. 2012, 2015), which like the context model cross-correlate the input with one or more filters (CFs), also are superior to multifilter LN models in describing complex cells. Hence, these previous results together with ours appear to highlight that cross-correlation model frameworks are better suited for describing complex cells on average.

The above finding still raises the question of why some complex cells were, nonetheless, better described by the multifilter LN model framework. One reason might be that simple and complex cells exist on a continuum (Mechler and Ringach 2002; Eickenberg et al. 2012). Also, complex cells are identified by exclusion (i.e., the virtue of not being simple cells), and hence, they constitute a diverse population (Martinez and Alonso 2003). Thus, it appears that this diversity is reflected in the variation of the models that best describe the stimulus-response mappings of complex cells. Consequently, some complex cells can be well described by a few filters only, while others require many. As models from cross-correlation frameworks, such as context models, provide a way of describing stimulus-response mappings which otherwise would require many LN model filters, one would expect these models to perform better for cells that actually need to be described with many filters. This is exactly what we observed, as the cells for which the multifilter LN models performed the best were also the ones with the highest correlation coefficients. That is, the multifilter LN models appeared to perform best for those few cells that could be best described with only two filters, whereas the context models were superior for the rest.

Finally, one needs to keep in mind that the performance difference between multifilter LN models and context models might be either smaller or larger than what was observed here. All of the best performing multifilter LN models (MID and MNE), as well as the context models, are estimated by finding a solution to a nonconvex optimization problem. For this reason, it is impossible to say whether the globally optimum model in each model framework was found. Thus, it is possible that better models might exist in each framework. However, an exhaustive search of the whole parameter space is not feasible. In practice, we are, therefore, restrained to comparing various proposed methods for estimating models from the two frameworks, which is what we have done here.

### What Is a Feature?

The single-CF context models resulted in a quantization of neural behavior, whereby the cell is driven by the pattern in the CF over a spatial range indicated by the RF. This resembles the hierarchical model of complex cells where these are thought to pool inputs from other cortical cells with similar but spatially shifted RFs (Antolík and Bednar 2011; Bair 2005; Chance et al. 1999; Hirsch and Martinez 2006; Hubel and Wiesel 1962; Martinez and Alonso 2003). In this view, the CF captures the spatially shifted RF of these input cells, as explicitly shown in the simulated network of this study (Fig. 4). The two-filter LN model, in contrast, describes the same insensitivity to the spatial shift of the RF of the input cells by using two filters (a quadrature pair), as also observed by others (Chen et al. 2007; Rust et al. 2005; Touryan et al. 2002). In this description, the filters do not correspond to the RF of any single input cell but, rather, to a linear combination of several shifted RFs.

The context and the two-filter LN models hence provide two different interpretations of what a feature is. Importantly, the current results indicate that for most complex cells, the context-model interpretation might be the more correct one. Although it is difficult to say why this should be, we suspect that each input cell’s RF can contribute to a relevant direction in stimulus space. The combined relevant subspace spanned by these directions is, hence, likely to be more than two-dimensional. Thus, a two-filter LN model, with its assumption of a two-dimensional subspace, would fail at describing the cell’s behavior, whereas a context model with a single CF might well be sufficient for this purpose.

### Intracortical Synaptic Depression

For 29% of the cells, the CFs of the baseline models exhibited a trailing negative tail with a spatial width of one bar. As illustrated in Westö and May (2016), such patterns cannot be explained by simple synaptic inhibition in the recorded cell, but rather reflect a situation where an excitatory pathway is temporarily unable to drive the recorded cell. In a hierarchical description where complex cells pool inputs from other cells, this would correspond to a recently activated input cell being unable to drive the complex cell. One potential explanation for this could be synaptic depression operating on the synapses connecting input cells to complex cells (Abbott and Regehr 2004; Abbott et al. 1997; Tsodyks and Markram 1997). Indeed, our previous simulations demonstrate that synaptic depression causes negative tails in CFs (Westö and May 2016). Alternatively, it is possible that the input cells or earlier cells along the visual pathway are experiencing local inhibition, although this inhibition would have to be very specific to produce a tail in the CF that is only one bar wide. Boudreau and Ferster (2005) observed a similar effect in the cat primary visual cortex, in cells that do not receive direct input from the thalamus. However, these authors were unable to establish whether the cause was synaptic depression or local inhibition.

### Nonstationary or Incorrect Mappings

We observed that I values could in some cases vary considerably between different training-testing configurations of the data. This could point toward nonmodeled adaptation effects and a subsequent model mismatch, whereby the stimulus-response function is simply not stationary in time or, alternatively, the tested models are unable to describe the cellsʼ true static stimulus response-mapping. Light might be shed on these issues by implementing online learning methods (Meyer et al. 2015), by modeling the suppressive surround region explicitly (Coen-Cagli et al. 2015; Goris et al. 2015), or by combining context models with other cross-correlation modeling frameworks. For example, the subunit models introduced by Eickenberg et al. (2012) and Vintch et al. (2012, 2015) can add additional flexibility in the form of multiple CFs or a nonlinearity that links the CFs with the RF. Nonetheless, context models have an advantage over both of these subunit implementations: the implementation in Eickenberg et al. (2012) naively assumes the equivalence of a uniform (or alternatively predefined) context model RF, whereas the implementation in Vintch et al. (2012, 2015) is trickier to estimate, as the optimization problem cannot be broken down into convex subproblems (as in the case of the context model). However, the context model is very closely related to the subunit model, and as it can be effectively estimated, it might turn out to provide valuable initial solutions for subunit models. A combined effort might, therefore, reveal the true reason behind the performance variation observed over the different training/testing configurations.

## GRANTS

This study was supported by the foundation for Aalto University Science and Technology Awards Grants, the Niilo Helander Foundation, the Otto A. Malm Foundation, and Svenska Kulturfonden (Finland). The calculations presented were performed using computer resources within the Aalto University School of Science “Science-IT” project. We are grateful for the support given by the MRC Institute of Hearing Research, University of Nottingham, UK, and by Special Laboratory Non-Invasive Brain Imaging at Leibniz Institute for Neurobiology, Magdeburg, Germany.

## DISCLOSURES

No conflicts of interest, financial or otherwise, are declared by the authors.

## AUTHOR CONTRIBUTIONS

J.W. and P.J.M. conceived and designed research; J.W. performed experiments; J.W. analyzed data; J.W. and P.J.M. interpreted results of experiments; J.W. prepared figures; J.W. and P.J.M. drafted manuscript; J.W. and P.J.M. edited and revised manuscript; J.W. and P.J.M. approved final version of manuscript.
